# Zinc status of northern Tasmanian adults

**DOI:** 10.1017/jns.2015.12

**Published:** 2015-04-20

**Authors:** Jeffrey M. Beckett, Madeleine J. Ball

**Affiliations:** School of Health Sciences, University of Tasmania, Launceston, Australia

**Keywords:** Zinc status, Population studies, Australia, AGP, α-1 acid glycoprotein, EAR, estimated average requirement, IZiNCG, International Zinc Nutrition Consultative Group

## Abstract

Information regarding Zn status in the Australian population is very limited. Mild deficiencies in Zn have been associated with CVD, impaired immune function and poor healing. A cross-sectional study of 497 northern Tasmanian adults (24–82 years of age) was conducted to assess Zn status. Dietary intakes were assessed by FFQ and serum concentrations of Zn were evaluated using International Zinc Nutrition Consultative Group methodology. Mean Zn intakes were 12·6 (sd 4·4) mg/d for men and 10·9 (sd 3·6) mg/d for women. It was found that 52 % of men but only 9 % of women consumed less than the Australia/New Zealand estimated average requirement for Zn. Mean serum Zn was 13·0 (sd 2·4) µmol/l in men and 13·0 (sd 2·5) µmol/l in women. Overall, 15 % of men and 7 % of women had low serum Zn levels. Furthermore, low serum Zn was observed in 18 % of men 50 years or older and 30 % of men 70 years or older. The present results suggest that mild Zn deficiency may be prevalent in older Tasmanian adults, particularly men; and due to the importance of Zn in many areas of health, this could be of public health concern.

Conservative estimates place approximately 20 % of the world's population at risk of Zn deficiency^(^^1^^)^. While it is a significant cause of morbidity in many developing countries, in developed countries information regarding the prevalence of Zn deficiency is more limited.

There is a paucity of data relating to the Zn status of Australians. As noted in a recent review of the literature concerning Zn status in Australia and New Zealand^(^^2^^)^, even in population groups considered ‘at risk’ there are few studies that have assessed Zn status. Published studies are generally limited by size, have sampled only specific population groups (for example, infants, institutionalised elderly populations) or only report Zn intakes^(^^2^^)^.

Studies that have assessed a broader range of healthy, community-dwelling adults in Australia have mostly reported Zn intakes only^(^^3^^–^^7^^)^, with few reporting serum Zn data^(^^8^^,^^9^^)^. As even the most recent of these Australian studies was reported a decade ago, they also pre-date the introduction of current procedures recommended by the International Zinc Nutrition Consultative Group (IZiNCG) for the assessment of Zn status; which include standardised collection procedures and age/sex/time of day-specific cut-off values for serum Zn^(^^10^^)^, making interpretation of the data difficult. Finding suitable biomarkers for Zn status in large study cohorts remains troublesome; various biomarkers have been investigated, such as leucocyte Zn^(^^11^^)^, lymphocyte ecto-5'nucleotidase^(^^12^^)^ as well as hair and urinary Zn. However, a 2009 systematic review suggests that serum Zn, when collected in controlled conditions, remains as perhaps the most convenient and reliable biomarker for assessing Zn status in population studies^(^^13^^)^. Serum Zn is sensitive to acute-phase responses, feeding state and time of day, and varies with sex and age. Collection systems are also vulnerable to contamination with exogenous Zn and so the methods outlined by IZiNCG are designed to control these various factors.

In addition to Zn intake, other dietary factors, such as meat and in particular the phytate content of the diet, can influence the bioavailability of Zn. Phytate has been observed to be a particular problem in developing countries in which diets are low in animal-based foods and rich in phytate-containing unrefined grains, cereals and legumes^(^^14^^)^. Although dietary fibre has also been thought to negatively affect Zn absorption it is likely that such a relationship was observed due to phytate generally being found in fibre-rich foods; research has shown that fibre in the absence of phytate has little influence on Zn absorption^(^^15^^)^. Even mild Zn deficiencies may be associated with increased morbidity, involving alterations in immune function^(^^16^^)^ and delayed wound healing^(^^17^^)^; Zn insufficiency has been associated with neural tube defects^(^^18^^)^ and is considered by the WHO as a candidate for prenatal programming of chronic disease in later life^(^^19^^)^.

Tasmania has a history of suboptimal or marginal status in other micronutrients such as iodine^(^^20^^)^ and Se^(^^21^^,^^22^^)^, but there is very little information about the status of other trace elements in the Tasmanian population. The population is ageing and has high rates of chronic disease^(^^23^^)^. Due to the lack of data relating to Zn status in Tasmanian populations, this cross-sectional study was undertaken to determine the Zn status of an adult population sample from northern Tasmania and identify any population groups that may be at risk of suboptimal status.

## Methods and materials

### Study population

This was a cross-sectional population study. The participants for the study were apparently healthy community-dwelling adults residing in the main population centres in north, northwest and northeastern Tasmania. Recruitment was by way of a mail-out to a random sample of adults taken from an extract of the Australian electoral roll provided by the Australian Electoral Commission. The study was conducted according to the guidelines laid down in the Declaration of Helsinki, and all procedures involving human subjects were approved by the Human Research Ethics Committee, Tasmania (reference no. H0009038). Written informed consent was obtained from all participants.

### Sample collection and preparation

Participants completed questionnaires to provide demographic and anthropometric information. A semi-quantitative FFQ^(^^24^^)^ which uses Australian food content tables, was used to collect dietary data. The socio-economic status of participants was estimated by using the SEIFA (Socio-Economic Indexes for Areas) index for the area (ABS Collector District) in which each subject resided. The SEIFA index is derived from Australian Census variables related to advantage and disadvantage, including households with low or high income, unemployment rates and proportions of individuals with limited or higher education^(^^25^^)^. A lower SEIFA score in a Census district indicates that the district is relatively disadvantaged compared with one with a higher score.

Participants attended phlebotomy centres to provide a morning, non-fasting, venous blood sample, collected into trace element-free Vacutainer tubes without anticoagulant (Becton Dickinson). Following collection, blood samples were separated by refrigerated centrifugation for 15 min at 1335 ***g***. Samples of serum were stored at –80°C until analysis. All laboratory glassware, consumables and storage vessels used during Zn analysis were acid washed (1 % HNO_3_) before use.

### Analytical methods

Serum Zn concentrations were determined by flame atomic absorption spectrometry using a Spectra 880 spectrophotometer (Varian Inc.) and the method of Meret & Henkin^(^^26^^)^. Analysis of Seronorm controls (Sero) with assayed Zn concentrations of 14·0 and 16·1 µmol/l gave means of 13·0 (CV 4·8 %; *n* 38) and 17·8 µmol/l (CV 4·0 %; *n* 38). Intra-assay precision was 3·5 % (*n* 13). Serum α-1 acid glycoprotein (AGP) was determined by an immunoturbidimetric method on a Konelab 20XT autoanalyser (Thermo Fisher Scientific) using Dako anti-AGP antibody. Analysis of Dako controls with AGP concentrations of 0·58 and 1·52 g/l gave means of 0·63 (CV 2·1 %; *n* 13) and 1·44 g/l (CV 1·8 %; *n* 13), respectively. Intra-assay precision was 2·2 % (*n* 20).

### Statistical analysis

Differences in Zn status within age and sex groups were estimated using general linear modelling (GLM) with robust standard error estimation (STATA version SE12; StataCorp LP). Post-estimation Holm's test analysis was used to adjust *P* values for multiple comparisons^(^^27^^)^. Associations between serum Zn and Zn intake, monounsaturated fat intake, age, socio-economic status and BMI were estimated using GLM. Selection of variables for inclusion in a multivariate model was performed using stepwise regression from: Zn intake, fat, protein and carbohydrate intake, age, socio-economic status, BMI and sex. The validity of regression assumptions was tested by *post hoc* analysis to exclude significant heteroskedasticity and missing variable effects.

Sample serum Zn concentrations were compared with the accepted sex/age cut-off values of 10·7 µmol/l for men and 10·1 µmol/l for women, as recommended by IZiNCG^(^^10^^)^. Most analyses used raw dietary intake data; however, as data were collected from a single time point, an adjusted estimate of the prevalence of low intakes was made, accounting for intra-individual variation, with an assumed CV of 25 %, as per IZiNCG's recommended methodology^(^^28^^)^.

As serum Zn is known to be reduced in subclinical infection or inflammation, serum AGP > 1·2 g/l was used as an indicator of current infection/inflammation. Due to the variation in response from different age and sex subgroups in the study sample, a population estimate was made using data from the 2006 Australian Census for this region, weighted for age, sex and socio-economic status.

## Results

### Subject characteristics

Subject characteristics are presented in Table 1. The study sample was comprised of 497 adults from northern Tasmania (191 men and 306 women) with a mean age of 57·4 (sd 12·3) years. Decadal age groups were 20–29 years (*n* 14), 30–39 years (*n* 36), 40–49 years (*n* 76), 50–59 years (*n* 128), 60–69 years (*n* 160), 70–79 years (*n* 81) and 80–89 years (*n* 2). Of the individuals who reported existing health conditions, the major conditions were hypertension (25 %) and arthritis (19 %); the most commonly reported medications used were anti-hypertensives (24 %), cholesterol-lowering drugs (16 %) and anticoagulants (12 %).

**Table 1.** Characteristics of the study subjects (Mean values, standard deviations and ranges)

	Male (*n* 191)			Female (*n* 306)		
	Mean	sd	Range	Mean	sd	Range
Age (years)	58·9	12·2	24–82	56·5	12·2	26–80
BMI (kg/m^2^)	27·7	4·6	18·5–43·8	26·5	5·2	15·9–46·8
Weight (kg)	86·2	15·1	57·0–155·0	70·6	14·8	46·0–132·0
Height (cm)	176·6	7·9	155·0–198·0	163·3	7·3	144·5–203·0
Dietary Zn (mg/d)*	12·6	4·4	4·0–29·6	10·9	3·6	3·5–33·4
Serum Zn (µmol/l)	13·0	2·4	7·5–24·5	13·0	2·5	7·6–23·8
Smoking (*n*)						
Never		96			185	
Previously		84			88	
Current		9			30	
Not known		2			3	

* Estimated average requirement: 12·0 mg/d for men; 6·5 mg/d for women^(^^22^^)^.

Mean contributions to total energy among the cohort for carbohydrate was 44 %, fats 34 % (saturated 14 %; monounsaturated 12 %; polyunsaturated 5 %) and protein 19 %. Major food sources of energy were dairy product-based foods 23 %, cereal-based foods 22 %, meat fish and poultry 14 % and fruit and vegetables 18 %. Mean dietary fibre was 26·7 g/d in men, and 21·1 g/d in women, less than the Australian adequate intake of 30 g/d and 25 g/d, respectively. The FFQ used in the present study did not specifically assess phytate levels and Australian food tables do not contain information on phytate content. Tasmanians, however, consume a modern Western diet in which phytate-rich food such as unleavened breads are rare and unrefined grains and seeds are consumed in relatively low amounts.

### Dietary zinc intake

Mean intakes of Zn were 12·6 (sd 4·4) mg/d for men and 10·9 (sd 3·6) mg/d for women. Overall, 26 % of the study sample consumed less than the Australian/New Zealand estimated average requirement (EAR) for Zn. Of the men, 52 % consumed less than the EAR for adult males (12 mg/d)^(^^29^^)^. The proportion of men that consumed less than the EAR increased with age. The lowest proportion was observed in the 20–29 years age range (20 %); increasing to 36 % (30–39 years), 46 % (40–49 years), 45 % (50–59 years), 54 % (60–69 years), 71 % (70–79 years) and 100 % in the 80–89 years age range.

Of the women, 9 % consumed less than the EAR for adult females of 6·5 mg/d. A trend for increasing proportions of low intakes with age was not observed in women; no women in the youngest or oldest age range consumed less than the EAR and the greatest proportion of women to do so were aged 40–49 years (12 %). Similar proportions of low intakes were observed in the remaining age ranges: 30–39 years, 8 %; 50–59 years, 10 %; 60–69 years, 8 %; 70–79 years, 8 %.

After adjusting for intra-individual variation (CV of 25 %)^(^^28^^)^, it was estimated that approximately 42·3 % of men and 5·4 % of women had Zn intakes below the EAR.

The major food groups contributing to Zn intakes were meat, fish and poultry, cereal products, legumes and nuts, vegetables and dairy products. Meat, fish and poultry made a significantly higher contribution to Zn intakes in men than in women (31·6 *v*. 26·3 %; 95 % CI of difference 3·1 to 7·2; *P* < 0·001), while vegetables (19·4 *v*. 16·8 %; 95 % CI of difference 1·2 to 3·9; *P* < 0·001), dairy products (20·1 *v*. 16·3 %; 95 % CI of difference 2·2 to 5·5; *P* < 0·001) and fruit (6·7 *v*. 5·2 %; 95 % CI of difference 0·8 to 2·1; *P* < 0·001) made greater contributions to Zn intakes for women.

Significantly higher vegetable consumption was observed in men older than 45 years compared with those younger than 45 years (359 *v*. 302 g; 95 % CI of difference 15 to 99; *P* = 0·008). This resulted in a significantly increased contribution to overall Zn intake from vegetables in men older than 45 years compared with younger men (17·9 *v*. 12·2 %; 95 % CI of difference 3·9 to 7·4; *P* < 0·001). The consumption of meat (132 *v*. 150 g; 95 % CI of difference –2 to 39; *P* = 0·07) and the proportion of Zn contributed by meat sources tended to be lower in the older men (31 *v*. 35 %; 95 % CI of difference –0·8 to 7·9; *P* = 0·11) but the differences did not reach statistical significance.

### Other associations with zinc status

Selection of measured variables associated with serum Zn using stepwise regression (Table 2) revealed positive associations with dietary Zn intake (*P* = 0·036) and BMI (*P* = 0·015), while monounsaturated fat intake, age and socio-economic status were negatively associated with serum Zn (all *P* < 0·02). There was no significant association between dietary fibre and serum Zn (*P* = 0·89).

**Table 2.** Multivariate analysis: association between mean serum zinc and variables selected by stepwise regression*

	Mean	β	95 % CI	*P*
Constant (µmol/l)†	13·0		12·8, 13·2	
Dietary Zn (mg/d)		0·3	0·1, 0·5	0·036
BMI (kg/m^2^)		0·3	0·1, 0·6	0·015
Dietary monounsaturated fat (g/d)		–0·4	–0·6, –0·1	0·009
Age (years)		–0·3	–0·5, –0·1	0·011
SEIFA‡		–0·3	–0·5, –0·1	0·006

SEIFA, Socio-Economic Indexes for Areas.

* The effect of dietary Zn intake, BMI, dietary monounsaturated fat, age and socio-economic status (SEIFA) (as standardised normal transformations) was estimated using general linear modelling. Variables were selected for this model from dietary Se intake, BMI, fat, protein and carbohydrate intake, age, sex and SEIFA, using stepwise regression.

† Constant is the overall mean serum Zn concentration.

‡ Derived from Australian Census variables related to advantage and disadvantage. A lower SEIFA score for a given population area indicates it is relatively disadvantaged compared with one with a higher score.

### Serum zinc

**Fig. 1.**
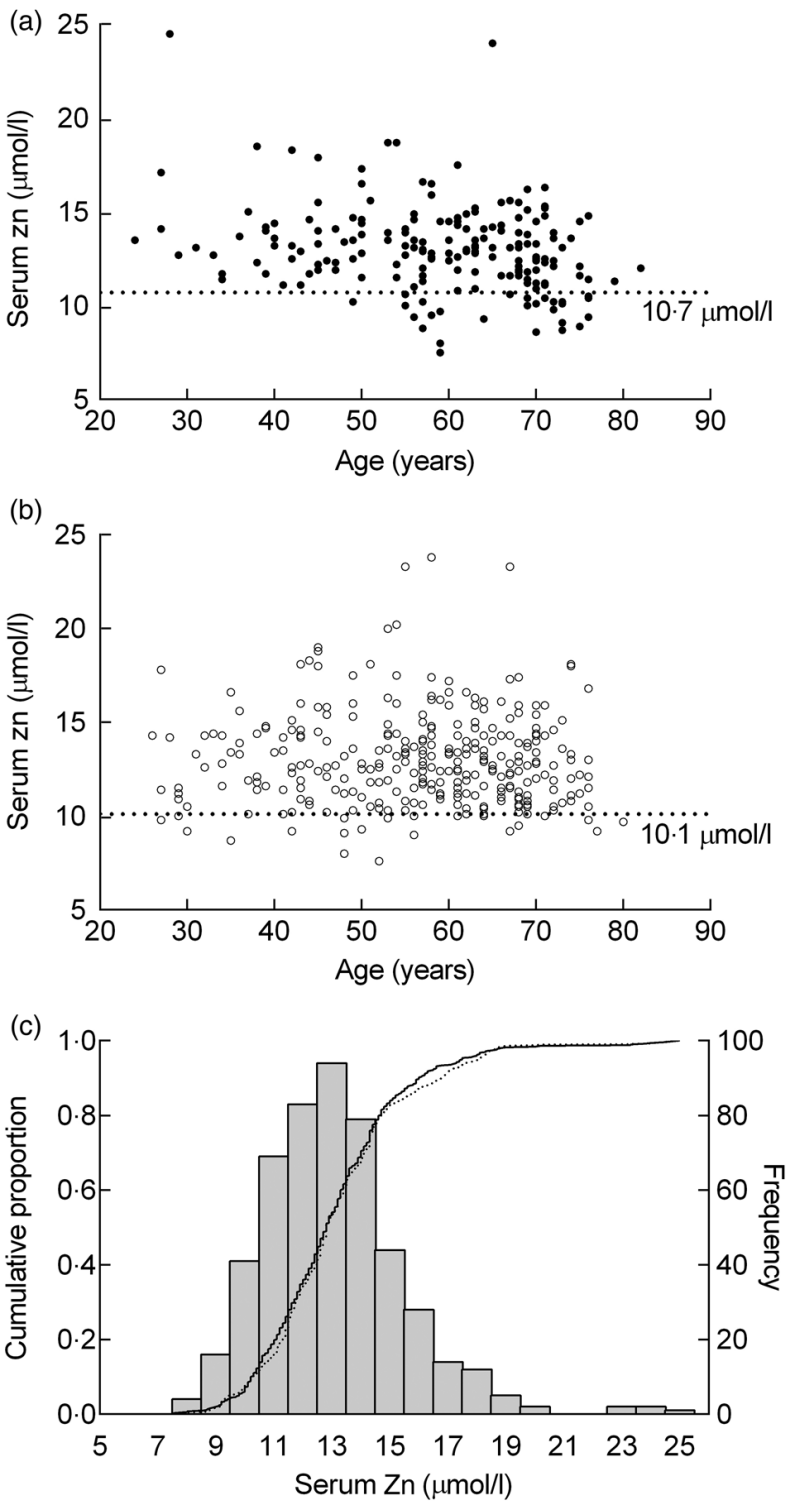
Serum zinc concentrations of population samples. (a) Serum zinc concentrations of male (*n* 191) and (b) female (*n* 306) subjects compared with International Zinc Nutrition Consultative Group (IZiNCG) cut-off values. (c) Frequency distribution (histogram), and cumulative proportion (lines) of sample (—) and population (····) estimate of serum zinc in northern Tasmania, adjusted for age, sex and socio-economic status.

Mean serum Zn was 13·0 (sd 2·4) and 13·0 (sd 2·5) µmol/l for men and women, respectively. Overall, serum Zn levels decreased with age in men, but not in women; this trend can be seen in Fig. 1(a) and (b). While 15 % of all men had low serum Zn, none aged <40 years had serum Zn below the IZiNCG cut-off (10·7 µmol/l)^(^^10^^)^. The prevalence of low serum Zn in men varied in the older age ranges; in the 40–49 years range only 4 % had serum Zn below the cut-off, but this increased to 19 % (50–59 years) and 8 % (60–69 years) and peaked at 31 % in those aged 70–79 years.

Of the women, 7 % had serum Zn below the IZiNCG cut-off (10·1 µmol/l)^(^^10^^)^. Of the women aged 20–29 years, 22 % had low serum Zn and while the only subject aged 80–89 years also had a low serum Zn level, the proportion in other age ranges was <10 %; 30–39 years (8 %), 40–49 years (8 %), 50–59 years (6 %), 60–69 years (4 %) and 70–79 years (8 %).

Of the participants, 3 % (*n* 15) had serum AGP > 1·2 g/l, indicating current inflammation/infection. Serum Zn was not significantly different in those who had AGP values below compared with above this level (12·1 *v*. 13·1 µmol/l; 95 % CI of difference –2·3 to 0·3; *P* = 0·13). Of these subjects with elevated AGP only two had serum Zn below cut-off values, one male in the 50–59 years age range and one female in the 30–39 years age range.

In the adjusted population estimates (Table 3 and Fig. 1(c)), serum Zn concentrations and the prevalence of low Zn intakes and serum Zn levels below relevant cut-off values were not significantly different compared with the study sample (*P* > 0·7).

**Table 3.** Estimates of population proportions with zinc intakes below the Australian/New Zealand estimated average requirement (EAR)* and with serum zinc below International Zinc Nutrition Consultative Group (IZiNCG) cut-offs†

	Sample estimate	95 % CI	Population estimate	95 % CI	Risk ratio‡	95 % CI	*P*
Zn intake							
Percentage < EAR	26	0·22, 0·30	25	0·21, 0·29	0·96	0·78, 1·19	0·72
Serum Zn							
Percentage < cut-off	9·9	0·07, 0·13	9·3	0·07, 0·12	0·94	0·64, 1·38	0·75

* Estimated average requirement: 12·0 mg/d for men; 6·5 mg/d for women^(^^22^^)^.

† IZiNCG serum Zn sex/age cut-off values: 10·7 µmol/l for men; 10·1 µmol/l for women^(^^10^^)^.

‡ Risk ratio (95 % CI) estimated by Poisson regression adjusted for age, sex and socio-economic status.

## Discussion

Micronutrient deficiency, including Zn deficiency, is a problem for a significant proportion of the world's population. Moreover, it appears that even the more commonly occurring mild deficiency can increase morbidity^(^^16^^,^^17^^,^^30^^)^, but there is very little information on the Zn status in Australian populations. In fact, the present study appears to be only the second to date to report both dietary and biochemical data on Zn status from a representative population sample in Australia, and the only such study in Tasmanians. The major finding is the relatively high prevalence of low Zn status in older Tasmanian men. In the present study over half of the men consumed less than the EAR for Zn (12 mg), and the prevalence of low intakes increased with age, with nearly 75 % of men over 70 years consuming inadequate Zn. Even in an adjusted estimate of intakes using a CV of 25 %, approximately 42 % of men reported low intakes. The prevalence of low serum Zn levels (<10·7 µmol/l) in older men was also high; low serum Zn was not observed in men below 40 years, but occurred in 18 % of men 50 years or older and 30 % of men 70 years or older. It is suggested that the risk of Zn deficiency in a population is elevated and of public health concern if >25 % of individuals consume inadequate intakes, or if >20 % have serum Zn below the relevant age/sex cut-off^(^^10^^)^.

Serum Zn concentrations can be decreased as an acute-phase response in inflammation and infection. Our assessment of serum AGP indicated that the prevalence of inflammation or infection in the population sample was low; this was not unexpected as participants had flexibility in when they could attend for phlebotomy and hence could delay providing blood samples if they were feeling unwell. Even if those with elevated AGP were excluded it would not change the interpretation of the study's results significantly; in fact it would slightly increase the proportion of both men and women with low serum Zn concentrations.

Our multivariate model indicated that the major influences on serum Zn status in this sample were dietary factors, BMI, age and socio-economic status. Interestingly, socio-economic status was negatively associated with serum Zn status, but while higher socio-economic status may often be considered to result in an improved diet quality and potentially higher micronutrient status, it has, in previous European studies, been associated with a slightly lower Zn status^(^^31^^)^.

In the present study, there were varying response rates in different population subgroups; however, the lack of difference between the sample and population estimates adjusted for age, sex and socio-economic status (Table 3) suggests that the overall findings from the sample are representative of the northern Tasmanian population.

Previous Australian studies of dietary Zn in adults have reported low intakes^(^^2^^,^^7^^)^; however, many are studies of aged-care residents. Of those to assess free-living older adults, results are conflicting. In a study of South Australian residents (in 1989; *n* 2195) aged 65+ years, Horwath^(^^7^^)^ reported relatively low mean intakes of 10·3 and 9·7 mg/d in men and women, respectively. Baghurst & Record^(^^32^^)^ (in 1987; *n* 331) also surveyed older South Australians (65–75 years) and reported somewhat higher median intakes of 12·0 mg/d in men and 10·9 mg/d in women, similar to the median intakes for individuals aged 65+ years in the present study of 11·1 mg/d (men) and 11·0 mg/d (women).

Overall, mean intakes in men from the present study (12·6 mg/d) were lower compared with the only other published Tasmanian data (13·5 mg/d) from the 1995 National Nutrition Survey^(^^5^^)^ and other Australian reports by English *et al.* (1983 National Dietary Survey of Adults; *n* 1974; 15·1 mg/d)^(^^3^^)^ and the 20th Australian Total Diet Survey (in 2003; 14 mg/d)^(^^4^^)^. Intakes were similar, however, to the large national study reported by Baghurst *et al.* (in 1991; *n* 806; 12·8 mg/d)^(^^9^^)^.

Women in the present study also consumed a similar Zn intake (10·9 mg/d) to that estimated by Baghurst *et al.* (in 1991; *n* 874; 11·2 mg/d)^(^^9^^)^ and English *et al.* (in 1983; *n* 2421; 10·5 mg/d)^(^^3^^)^ but approximately 20 % more than Tasmanian women in the 1995 National Nutrition Survey (9·1 mg/d)^(^^5^^)^ and 30 % more than estimated in the 20th Australian Total Diet Survey (in 2003; 8·4 mg/d)^(^^4^^)^. While the present study, like those by Horwath^(^^7^^)^ and Baghurst *et al.*^(^^9^^)^, utilised semi-quantitative FFQ for dietary estimates, methodological differences from many of the other previous studies make further comparisons inappropriate.

The study of Baghurst *et al.*^(^^9^^)^ also determined serum Zn in a smaller sample of South Australian adults, reporting mean values of 15·1 µmol/l (*n* 90) and 14·1 µmol/l (*n* 111) for men and women, respectively. This study preceded the use of IZiNCG cut-off values^(^^10^^)^ for assessing the risk of Zn deficiency and hence did not calculate the proportions of individuals below the now-accepted cut-off values. The authors, however, did note a trend of decreasing serum Zn values with age, which, as in the present study, was most evident in men.

Comparable serum Zn data from healthy adult populations in other countries also appear limited. Similar serum Zn levels have been reported by one UK study^(^^33^^)^ in men (13·9 µmol/l; *n* 95) and women (13·3 µmol/l; *n* 94) with mean participant ages (45 and 49 years, respectively) lower than the present study. Serum Zn was also similar in a large Northern Ireland study^(^^34^^)^ in both men (13·2 µmol/l; *n* 1142) and women (12·7 µmol/l; *n* 1034); this study also reported a decline in Zn status with age among men, but not women.

A New Zealand study^(^^35^^)^ of elderly women (mean age 74·9 years; *n* 102) reported a mean serum Zn of 12·4 µmol/l. As noted by the authors, this concentration is relatively low compared with other published data, but it was still higher than women of a similar age in the present study (11·6 µmol/l).

A lower Zn status in older age groups is in accordance with a number of other studies^(^^2^^,^^34^^–^^36^^)^, and is a trend possibly due to a decrease in gastrointestinal absorption efficiency with age^(^^37^^)^, but changes in diet, as seen in the present study, as well as social and economic factors are also likely contributors. Zn is important for many broad biological processes. Recent research^(^^36^^)^ indicates an association between low Zn status and decreased immune function in the elderly and that low Zn status may be an important contributor to the immunosenescence observed in old age. Zn has also been associated with CVD, diabetes, neurodegenerative disease and cancer^(^^38^^)^. Of the cancers, the development and progression of prostate cancer in particular appears to be associated with low Zn levels^(^^39^^)^, which in the context of this present study's findings, may be important.

The present results suggest that low Zn status may occur in a significant proportion of community-dwelling Tasmanians, and that there could be a relatively high prevalence of low Zn status in older males. Previous studies have shown that low Zn status is common in residential aged-care populations elsewhere in Australia^(^^2^^)^, and therefore the prevalence of low Zn status across the entire older Tasmanian population may be greater than suggested by the present study.

In the present study, the lack of agreement between the proportions of male subjects estimated to consume inadequate Zn and those with low serum Zn concentrations was probably due to a combination of factors, including limitations of the dietary data collection method (FFQ), intra-individual variability and under-reporting. The combined effect of these factors may explain why previous observational studies have generally failed to find significant associations between Zn intakes and serum Zn concentrations^(^^40^^)^. Established knowledge about the phytate content of Australian diets^(^^41^^)^, and the dietary information collected in the present study suggest that phytate intake is likely to have minimal influence on Zn status in this population.

The measurement of dietary intakes and serum Zn on multiple occasions for each participant would have strengthened the present study and should be included in future studies in this area. However, the findings of the study provide useful insight into groups that may be at risk of mild Zn deficiency in a population where no other data have been reported.

While the study sample had relatively low numbers of subjects in the extremes of the age range (youngest and oldest) there was reasonable representation of most age ranges and in particular those where the most significant findings were made (50+ years; *n* 363). Further study of middle-aged and elderly Tasmanians, including both community-dwelling and aged-care residents, would be beneficial to confirm the prevalence of mild Zn deficiency in this population. Further investigation of the Zn status in the older populations of other states of Australia may also be warranted.

## References

[1] Sanghvi T, Van Ameringen M, Baker J, (2007) Vitamin and mineral deficiencies technical situation analysis: a report for the Ten Year Strategy for the Reduction of Vitamin and Mineral Deficiencies. Food Nutr Bull 28, S160–S219. 17674508

[2] Gibson R & Heath A-L (2011) Population groups at risk of zinc deficiency in Australia and New Zealand. Nutr Diet 68, 97–108.

[3] English RM, Najman JM & Bennett SA (1997) Dietary intake of Australian smokers and nonsmokers. Aust N Z J Public Health 21, 141–146. 9161068 10.1111/j.1467-842x.1997.tb01673.x

[4] Food Standards Australia New Zealand (2003) 20th Australian Total Diet Survey. Canberra: Food Standards Australia New Zealand.

[5] McLennan W & Podger A (1995) National Nutrition Survey: Nutrient Intakes and Physical Measurements. Canberra: Australian Bureau of Statistics.

[6] McCarty CA, Nanjan MB & Taylor HR (2002) Dietary intake of older Victorians. Nutr Diet 59, 12–17.

[7] Horwath CC (1989) Dietary survey of a large random sample of elderly people: energy and nutrient intakes. Nutr Res 9, 479–492.

[8] Ball MJ & Ackland ML (2000) Zinc intake and status in Australian vegetarians. Br J Nutr 83, 27–33. 10703461 10.1017/s0007114500000052

[9] Baghurst KI, Dreosti IE, Syrette JA, (1991) Zinc and magnesium status of Australian adults. Nutr Res 11, 23–32.

[10] Brown KH, Rivera JA, Bhutta Z, (2004) International Zinc Nutrition Consultative Group (IZiNCG) technical document #1. Assessment of the risk of zinc deficiency in populations and options for its control. Food Nutr Bull 25, S99–S203. 18046856

[11] Whitehouse RC, Prasad AS, Rabbani PI, (1982) Zinc in plasma, neutrophils, lymphocytes, and erythrocytes as determined by flameless atomic absorption spectrophotometry. Clin Chem 28, 475–480. 7067090

[12] Meftah S, Prasad AS, Lee DY, (1991) Ecto 5' nucleotidase (5′NT) as a sensitive indicator of human zinc deficiency. J Lab Clin Med 118, 309–316. 1940572

[13] Lowe NM, Fekete K & Decsi T (2009) Methods of assessment of zinc status in humans: a systematic review. Am J Clin Nutr 89, 2040S–2051S. 19420098 10.3945/ajcn.2009.27230G

[14] Schlemmer U, Frolich W, Prieto RM, (2009) Phytate in foods and significance for humans: food sources, intake, processing, bioavailability, protective role and analysis. Mol Nutr Food Res 53, Suppl. 2, S330–S375. 19774556 10.1002/mnfr.200900099

[15] Lonnerdal B (2000) Dietary factors influencing zinc absorption. J Nutr 130, 1378S–1383S. 10801947 10.1093/jn/130.5.1378S

[16] Ibs KH & Rink L (2003) Zinc-altered immune function. J Nutr 133, 1452S–1456S. 12730441 10.1093/jn/133.5.1452S

[17] Lansdown ABG, Mirastschijski U, Stubbs N, (2007) Zinc in wound healing: theoretical, experimental and clinical aspects. Wound Repair Regen 15, 2–16. 17244314 10.1111/j.1524-475X.2006.00179.x

[18] Velie EM, Block G, Shaw GM, (1999) Maternal supplemental and dietary zinc intake and the occurrence of neural tube defects in California. Am J Epidemiol 150, 605–616. 10490000 10.1093/oxfordjournals.aje.a010059

[19] Delisle H (2002) Programming of Chronic Disease by Impaired Fetal Nutrition. Evidence and Implications for Policy and Intervention Strategies. Geneva: WHO, Department of Nutrition for Health and Development.

[20] Guttikonda K, Burgess JR, Hynes K, (2002) Recurrent iodine deficiency in Tasmania, Australia: a salutary lesson in sustainable iodine prophylaxis and its monitoring. J Clin Endocrinol Metab 87, 2809–2815. 12050255 10.1210/jcem.87.6.8600

[21] McGlashan ND, Cook SJ, Melrose W, (1996) Maternal selenium levels and sudden infant death syndrome (SIDS). Aust N Z J Med 26, 677–682. 8958364 10.1111/j.1445-5994.1996.tb02939.x

[22] Beckett JM & Ball MJ (2011) Marginal selenium status in northern Tasmania. Br J Nutr 106, 718–724. 21521546 10.1017/S0007114511000687

[23] Australian Bureau of Statistics (2006) National Health Survey: Summary of Results (2004–2005). Canberra: Australian Bureau of Statistics.

[24] Giles GG & Ireland PD (1996) Dietary Questionnaire for Epidemiological Studies (Version 2). Melbourne: The Cancer Council Victoria.

[25] Australian Bureau of Statistics (2006) Socio-Economic Indexes for Areas (SEIFA): Technical Paper. Canberra: Australian Bureau of Statistics.

[26] Meret S & Henkin RI (1971) Simultaneous direct estimation by atomic absorption spectrophotometry of copper and zinc in serum, urine, and cerebrospinal fluid. Clin Chem 17, 369–373. 5573399

[27] Aickin M & Gensler H (1996) Adjusting for multiple testing when reporting research results: the Bonferroni vs Holm methods. Am J Public Health 86, 726–728. 8629727 10.2105/ajph.86.5.726 PMC1380484

[28] International Zinc Nutrition Consultative Group (IZiNCG) (2007) Determining the Prevalence of Zinc Deficiency: Assessment of Dietary Zinc Intake. Technical Brief 3. Davis, CA: International Zinc Nutrition Consultative Group, c/o Program in International and Community Nutition, University of California.

[29] National Health and Medical Research Council (2006) Nutrient Reference Values for Australia and New Zealand. Canberra: National Health and Medical Research Council.

[30] MacDonald RS (2000) The role of zinc in growth and cell proliferation. J Nutr 130, 1500S–1508S. 10801966 10.1093/jn/130.5.1500S

[31] Novaković R, Cavelaars A, Geelen A, (2014) Socio-economic determinants of micronutrient intake and status in Europe: a systematic review. Public Health Nutr 17, 1031–1045. 23750829 10.1017/S1368980013001341 PMC10282449

[32] Baghurst KI & Record SJ (1987) The vitamin and mineral intake of a free-living young elderly Australian population in relation to total diet and supplementation practices. Hum Nutr Appl Nutr 41, 327–337. 3692899

[33] Ghayour-Mobarhan M, Taylor A, New SA, (2005) Determinants of serum copper, zinc and selenium in healthy subjects. Ann Clin Biochem 42, 364–375. 16168192 10.1258/0004563054889990

[34] McMaster D, McCrum E, Patterson CC, (1992) Serum copper and zinc in random samples of the population of Northern Ireland. Am J Clin Nutr 56, 440–446. 1636623 10.1093/ajcn/56.2.440

[35] De Jong N, Gibson RS, Thomson CD, (2001) Selenium and zinc status are suboptimal in a sample of older New Zealand women in a community-based study. J Nutr 131, 2677–2684. 11584090 10.1093/jn/131.10.2677

[36] Haase H & Rink L (2009) The immune system and the impact of zinc during aging. Immun Ageing 6, 9. 19523191 10.1186/1742-4933-6-9 PMC2702361

[37] Russell RM (1992) Changes in gastrointestinal function attributed to aging. Am J Clin Nutr 55, 1203S–1207S. 1590257 10.1093/ajcn/55.6.1203S

[38] Chasapis CT, Loutsidou AC, Spiliopoulou CA, (2012) Zinc and human health: an update. Arch Toxicol 86, 521–534. 22071549 10.1007/s00204-011-0775-1

[39] Costello LC & Franklin RB (2011) Zinc is decreased in prostate cancer: an established relationship of prostate cancer! J Biol Inorg Chem 16, 3–8. 21140181 10.1007/s00775-010-0736-9 PMC3735606

[40] Lowe NM, Medina MW, Stammers AL, (2012) The relationship between zinc intake and serum/plasma zinc concentration in adults: a systematic review and dose–response meta-analysis by the EURRECA Network. Br J Nutr 108, 1962–1971. 23244547 10.1017/S0007114512004382

[41] International Zinc Nutrition Consultative Group (IZiNCG) (2004) International Zinc Nutrition Consultative Group (IZiNCG) technical document #1. Assessment of the risk of zinc deficiency in populations and options for its control. Food Nutr Bull 25, S95–S203. 18046856

